# Supplementary material for: SemiBin2: self-supervised contrastive learning leads to better MAGs for short- and long-read sequencing
Source: Bioinformatics. 2023 Jun 30;39(Suppl 1):i21–9. doi: 10.1093/bioinformatics/btad209 (PMC10311329; doi:10.1093/bioinformatics/btad209)
Supplement: btad209_Supplementary_Data [file btad209_supplementary_data.pdf]

SemiBin2: self-supervised contrastive learning leads to better MAGs for short- and long-read sequencing

Shaojun Pan [1,2], **Xing-Ming Zhao** [1,2,3,4,*], **and Luis Pedro Coelho** [1,2,*]

[1]Institute of Science and Technology for Brain-Inspired Intelligence, Fudan University, Shanghai, China.

[2]Key Laboratory of Computational Neuroscience and Brain-Inspired Intelligence, Ministry of Education, Shanghai, China.

[3]MOE Frontiers Center for Brain Science, Fudan University, Shanghai, China.

[4]Zhangjiang Fudan International Innovation Center, Shanghai, China.

[*]to whom correspondence should be addressed: xmzhao@fudan.edu.cn and luispedro@big-data-biology.org

1 Supplementary Figures and Tables

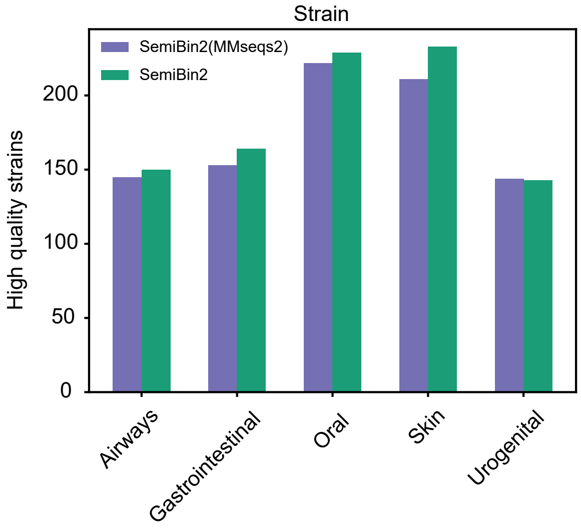

Supplementary Fig 1. Self-supervised learning achieved better binning results compared to semi-supervised in CAMI II long-reads sequencing datasets. To compare the semi-supervised to self-supervised learning, we used their embeddings with the same ensemble-based DBSCAN clustering method in CAMI II long-reads sequencing datasets. Self-supervised learning achieved similar or better binning results compared to semi-supervised learning. SemiBin2(MMseqs2): using embeddings from semi-supervised learning; SemiBin2: using embeddings from self-supervised learning. Shown is the number of distinct high-quality strains.

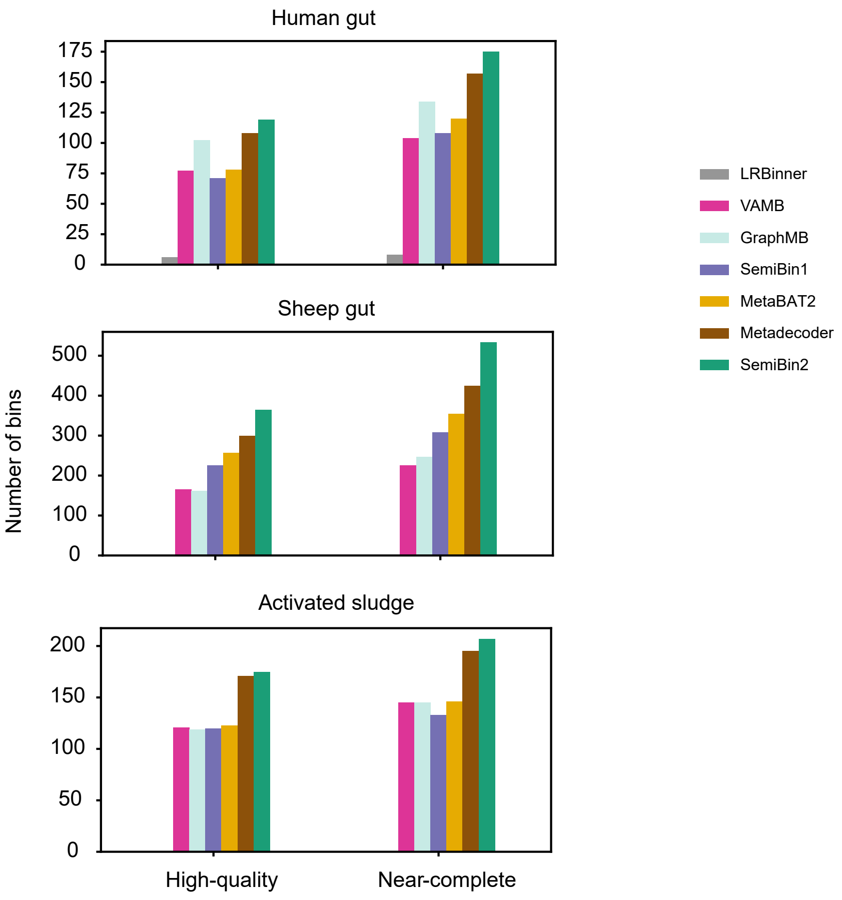

Supplementary Fig 2. SemiBin2 outperformed other binners on real long-read datasets when performance was evaluated with CheckM1. As noted in the main text, because of the use of an overlapping set of genes for generating the bins and evaluation, this is not the strictest evaluation model.

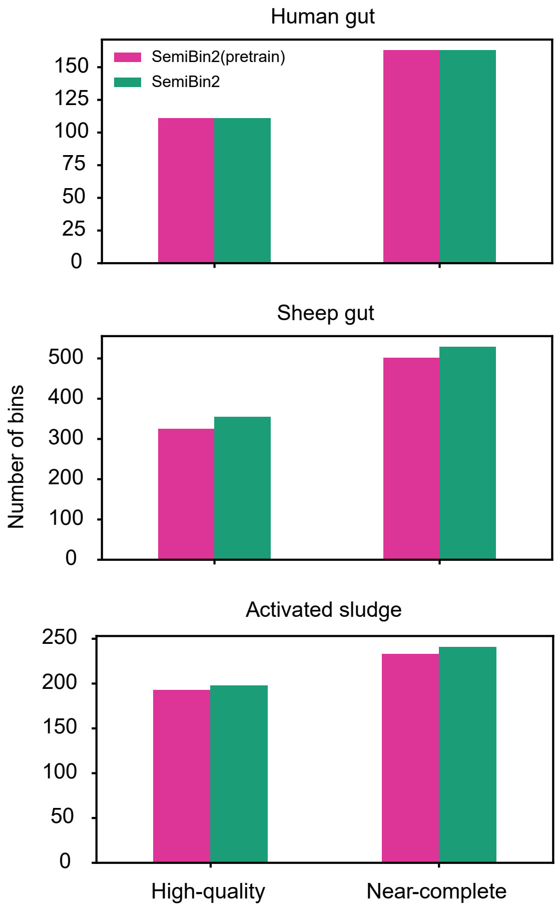

Supplementary Fig 3. SemiBin2(pretrain) could also get good binning results on real long-read datasets. To test the performance of pretrained models on long-read datasets, we compared SemiBin2 (model trained from every sample) to SemiBin2(pretrain), which used the pretrained models already developed for SemiBin1[Pan *et al.*(2022)]. For human gut dataset, we used the `human_gut` pretrain model and for other two projects, we used `global` pretrain model. Note that these pretrained models were built from short-read samples, they still produced good binning results on long-read datasets.

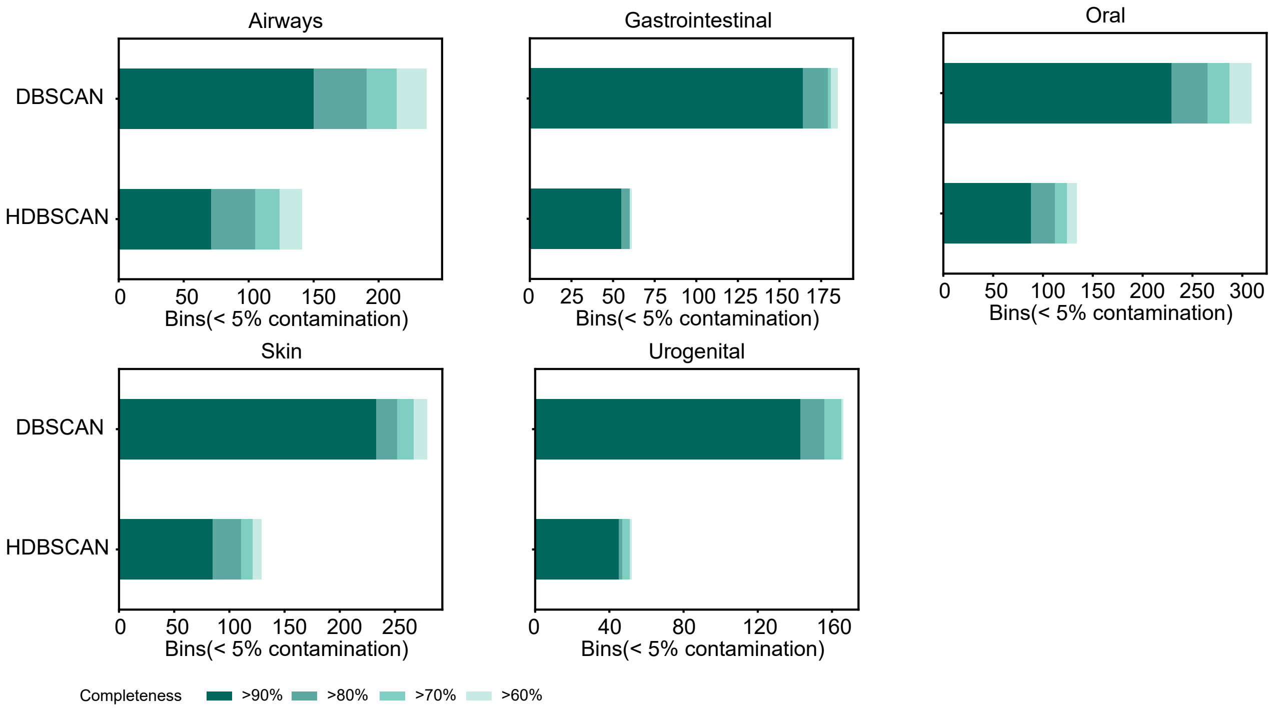

Supplementary Fig 4. SemiBin2 with DBSCAN outperformed HDBSCAN in CAMI II long-read sequencing datasets. We compared another clustering algorithm HDBSCAN[Campello *et al.*(2013)] (https://github.com/scikit-learn-contrib/hdbscan) to DBSCAN algorithm used in SemiBin2. We run HDBSCAN with *min_cluster_size* equals to 2,3,5,10,15,20,30,50,100,120,150,200 (the *min_cluster_size* must >1) and integrated these results using the same greedy algorithm. The results shown that ensemble-based DBSCAN algorithm performed better than ensemble-based HDBSCAN algorithm, indicting the HDBSCAN method might not reconstruct small size of bins (will be left as noise) and not suitable for binning long-read sequencing datasets (some genomes consisting of few contigs, even a single contig).

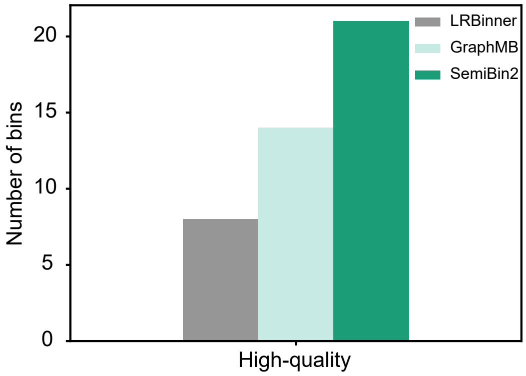

Supplementary Fig 5. SemiBin2 outperformed GraphMB and LRBinner in simulated long-read sequencing dataset. As we could not compare SemiBin2 to GraphMB and LRBinner in CAMI II long-read sequencing datasets. We simulated a long-read dataset with CAMISIM[Fritz *et al.*(2019)] based on strains from CAMI II. SemiBin2 reconstructed most high-quality bins compared to GraphMB and LRBinner in this dataset.

		#contig	Average length	N50
Sample A	mNGS	277,884	1,865	8,778
	PacBio-HiFi	2,575	99,842	269,406
	Nanopore	3,414	65,685	199,639
Sample B	mNGS	146,857	2,402	28,310
	PacBio-HiFi	1,447	157,228	1,081,788
	Nanopore	1,795	123,512	658,841
Sample C	mNGS	170,813	1,956	18,485
	PacBio-HiFi	822	171,215	1,270,126
	Nanopore	1,370	126,533	891,411

Supplementary Table 1. Statistics of assemblies from mNGS, PacBio-HiFi and Nanopore sequencing of 3 human gut samples. #contig: Number of contigs. Average length: average length of contigs. N50: the length of the shortest contig at 50% of the total assembly length.

		Taxonomic annotation			Randomly sampling		
		Nr. Cannot	Acc %	Nr. Covered	Nr. Cannot	Acc %	Nr. Covered
mNGS (multi-sample binning)	Airways	35,772,061	99.64	885	39,007,000	96.81	1085
	GI	25,628,395	98.67	291	28,843,000	92.23	511
	Oral	37,794,493	99.75	1,135	40,000,000	98.19	1,393
	Skin	21,908,477	99.47	614	25,814,000	95.71	822
	Urog	15,229,369	98.61	271	18,710,000	93.03	408
PacBio (co-assembly binning)	Airways	4,000,000	99.96	806	4,000,000	99.68	934
	GI	4,000,000	99.74	128	4,000,000	97.13	281
	Oral	4,000,000	99.95	654	4,000,000	99.54	799
	Skin	4,000,000	99.92	569	4,000,000	99.39	725
	Urog	4,000,000	99.81	192	4,000,000	98.25	300

Supplementary Table 2. Accuracy of cannot-link constraints generated using MMseqs2 and randomly sampling in CAMI II simulated datasets. GI: Gastrointestinal. Urog: Urogenital. Nr. Cannot(MMseqs2): number of cannot-link constraints generated from MMseqs2. Acc(MMseqs2): the accuracy of cannot-link constraints generated from MMseqs2. Nr. Covered(MMseqs2): the number of genomes that are covered by the accurate cannot-link constraints generated from MMseqs2. Nr. Cannot(random), Acc(random) and Nr. Covered(random) are the corresponding results generated by randomly sampling.

	Datasets	#Samples	#Genomes	Sequencing	Binning mode
CAMI II	Airways	10	828	mNGS	multi-sample
	GI	10	268	mNGS	multi-sample
	Oral	10	799	mNGS	multi-sample
	Skin	10	610	mNGS	multi-sample
	Urog	9	254	mNGS	multi-sample
CAMI II	Airways	10	935	PacBio	co-assembly
	GI	10	281	PacBio	co-assembly
	Oral	10	799	PacBio	co-assembly
	Skin	10	725	PacBio	co-assembly
	Urog	9	301	PacBio	co-assembly
Real datasets	Human gut	82	Unknown	mNGS	multi-sample
	Dog gut	129	Unknown	mNGS	multi-sample
	Ocean	109	Unknown	mNGS	multi-sample
	Soil	101	Unknown	mNGS	multi-sample
PRJCA007414	SAMC515478	1	UnKnown	PacBio-HiFi	single-sample
	SAMC515479	1	UnKnown	PacBio-HiFi	single-sample
	SAMC515480	1	UnKnown	PacBio-HiFi	single-sample
	SAMC515478	1	UnKnown	Nanopore R9.4	single-sample
	SAMC515479	1	UnKnown	Nanopore R9.4	single-sample
	SAMC515480	1	UnKnown	Nanopore R9.4	single-sample
PRJNA595610	SRR10963010	1	UnKnown	PacBio-HiFi	single-sample
	SRR14289618	1	UnKnown	PacBio-HiFi	single-sample
PRJEB48021	SAMEA9994818	1	UnKnown	PacBio-HiFi	single-sample
	SAMEA9994819	1	UnKnown	NanoPore R9.4.1	single-sample
	SAMEA9994820	1	UnKnown	NanoPore R9.4.1	single-sample
	SAMEA9994714	1	UnKnown	NanoPore R10.4	single-sample

Supplementary Table 3. Overview of the datasets used in the benchmarking. GI: Gastrointestinal. Urog: Urogenital.

		Time(min)			Memory(MB)		
		Human gut	Dog gut	Ocean	Human gut	Dog gut	Ocean
Multi-sample binning	VAMB(CPU)	10.3	10.6	17.9	1,085	1,291	918
	VAMB(GPU)	1.7	2.2	3.0	3,690	3,880	3,495
	SemiBin1(CPU)	276.5	297.3	341.0	39,070	37,904	46,091
	SemiBin1(GPU)	126.3	122.8	164.0	39,070	37,904	46,091
	SemiBin2(CPU)	142.9	163.1	171.9	3,887	3,655	4,775
	SemiBin2(GPU)	30.4	32.6	39.2	4,491	4,364	5,233

Supplementary Table 4. Running time and memory usage for the different binning tools with multi-sample binning. Average per sample time and peak memory usage (over 10 randomly chosen samples). For CPU timings, we used an AWS g4ad.4xlarge machine with 1 CPU, 8 physical cores and 16 logical cores. For GPU machine, we used a Tesla T4. GPU: Graphical Processing Unit, CPU: Central Processing Unit.

Supplementary References

Pan *et al.*(2022). A deep siamese neural network improves metagenome-assembled genomes in microbiome datasets across different environments. *Nature Communications*, **13**(1), 1–12.

Campello *et al.*(2013). Density-based clustering based on hierarchical density estimates. In *Advances in Knowledge Discovery and Data Mining: 17th Pacific-Asia Conference, PAKDD 2013, Gold Coast, Australia, April 14-17, 2013, Proceedings, Part II 17*, pp. 160–172. Springer.

Fritz *et al.*(2019). Camisim: simulating metagenomes and microbial communities. *Microbiome*, **7**(1), 1–12.
